# The Role of Sirtuins in the Pathogenesis of Psoriasis

**DOI:** 10.3390/ijms241310782

**Published:** 2023-06-28

**Authors:** Sylwia Słuczanowska-Głabowska, Maria Salmanowicz, Marzena Staniszewska, Andrzej Pawlik

**Affiliations:** Department of Physiology, Pomeranian Medical University, 70-204 Szczecin, Poland; sglabowska@gmail.com (S.S.-G.); maria.salmanowicz@gmail.com (M.S.); marzena.staniszewska@pum.edu.pl (M.S.)

**Keywords:** psoriasis, sirtuins, SIRT1, oxidative stress, dermatology

## Abstract

Psoriasis is the most common chronic inflammatory skin disease with a genetic basis. It is characterised by keratinocyte hyperproliferation, parakeratosis and inflammatory cell infiltration. Psoriasis negatively affects a patient’s physical and emotional quality of life. Sirtuins (SIRTs; silent information regulators) are an evolutionarily conserved group of enzymes involved in the post-translational modification of proteins, including deacetylation, polyADP-ribosylation, demalonylation and lipoamidation. SIRTs are involved in a number of cellular pathways related to ageing, inflammation, oxidative stress, epigenetics, tumorigenesis, the cell cycle, DNA repair and cell proliferation, positioning them as an essential component in the pathogenesis of many diseases, including psoriasis. Activation of SIRT1 counteracts oxidative-stress-induced damage by inhibiting the mitogen-activated protein kinases (MAPK), nuclear factor kappa-light-chain-enhancer of activated B cells (NF-κB) and signal transducer and activator of transcription 3 (STAT3) pathways and may mitigate pathological events in psoriasis. There is a significant reduction in the expression of SIRT1, SIRT2, SIRT3, SIRT4 and SIRT5 and an increase in the expression of SIRT6 and SIRT7 in psoriasis. The aim of the review is to draw the attention of physicians and scientists to the importance of SIRTs in dermatology and to provide a basis and impetus for future discussions, research and pharmacological discoveries to modulate SIRT activity. In light of the analysis of the mode of action of SIRTs in psoriasis, SIRT1–SIRT5 agonists and SIRT6 and SIRT7 inhibitors may represent new therapeutic options for the treatment of psoriasis.

## 1. Introduction

### 1.1. Psoriasis—Epidemiology

Psoriasis is a common skin disease with a genetic basis. It is estimated to affect about 1.5–3% of the population worldwide. It is present in all geographic regions and in people of different races. It occurs more often in highly developed countries, more often in Europe than in America, more often in the Caucasian race than in other races and with the same frequency in men and women. It is a chronic, non-infectious and relapsing disorder that clinically manifests as papular lesions covered with silvery scales [1].

About 30% of patients with psoriasis have a family history of the disease, and the inheritance is described as polygenic and autosomal-dominant with limited penetrance. The risk of a child developing psoriasis in a healthy family is about 1–2%, while it rises to 10–20% if one parent has the disease. If both parents have the disease, the risk can reach 50–70%. The incidence of the disease is often noted equally in men and women. There are two peaks of incidence at the ages of 20 and 60 years. There are two types of psoriasis, depending on the age of onset. Type I psoriasis affects people diagnosed before the age of 40 years; has a strong association with the HLA-Cw6, B13 and B57 antigens; and tends to have a familial autosomal-dominant inheritance. Type I psoriasis is characterised by early onset, frequent exacerbations and a tendency towards severe courses. Type II psoriasis is most common between the ages of 50 and 70 years and is associated with HLA-Cw2, Cw6 and B27 antigens, but it is less common in families. As a rule, the course is stable and skin lesions are more limited [2].

The primary skin lesion is papules covered with silvery scales, and the lesions localise on both smooth skin and the scalp. More than 85% of patients have plaque psoriasis. Other forms include palmoplantar psoriasis, inverted psoriasis, pustular psoriasis and scaly erythroderma [3]. Generalised pustular psoriasis is the most severe form of the disease and is life-threatening [2]. Psoriatic arthritis affects a quarter of patients with psoriasis. Psoriasis is a disease that significantly affects a patient’s physical, mental and emotional quality of life [3].

The global and regional incidence of psoriasis was assessed in the Global Burden of Disease (GBD) study [4]. Between 1990 and 2019, the global incidence of psoriasis increased by 26.53%, but the age-standardized incidence rate of psoriasis (ASIR) decreased. Regionally, the largest decrease in the ASIR of psoriasis between 1990 and 2019 was observed in central and eastern sub-Saharan Africa. An increase in the ASIR of psoriasis was observed only in Japan.

This study also assessed trends in the prevalence of seborrheic dermatitis [5]. Over the years, from 1990 to 2019, the prevalence of seborrheic dermatitis showed a slow upward trend. In 2019, the regions with the highest prevalence worldwide were sub-Saharan Africa and North America, while Central Asia and Eastern Europe had the lowest prevalence of seborrheic dermatitis.

### 1.2. Psoriasis—Aetiopathogenesis

The basic phenomenon in psoriasis is hyperkeratosis, or excessive epidermal proliferation, and parakeratosis, the essence of which is an abnormal differentiation of keratinocytes characterised by the presence of cell nuclei in the stratum corneum. The aetiopathogenesis of psoriasis is still not fully understood. Environmental factors including, but not limited to, infection, trauma and drugs, as well as genetic and immunological factors, are involved in its development. Researchers have shown that plasmacytoid dendritic cells (pDCs) play a major role in the initial phase of psoriasis. The mechanism of pDC activation is not fully understood. It probably results from the cooperation between superantigens and unknown autoantigens present in the epidermis, keratinocytes and T lymphocytes. As a result of pDC activation, there is a stimulation of keratinocytes and T lymphocytes, especially Th17, and a reduction in the number of antigen-presenting cells (including Langerhans cells) that migrate into the skin as part of the inflammatory infiltration. Activation of keratinocytes and lymphocytes is crucial for the formation of psoriatic lesions. An increase in the expression of transforming growth factor alpha (TGF-α) and ligands for the epidermal growth factor (EGF) receptor in the skin is also characteristic [4,5]. Multiple vascular changes, including increased angiogenesis, are also observed in the development of psoriatic lesions [1].

Psoriatic skin lesions are characterised by the presence of diverse populations of inflammatory cells, such as T cells, B cells, neutrophils and DCs. These cells release a number of cytokines such as interleukin 1 (IL-1), IL-2, IL-6, IL-8, IL-12, IL-17, IL-18, IL-22, IL-23, TNF-α, interferon gamma (IFN-γ) and transforming growth factor beta (TGF-ß), among others. These cytokines, especially IL-23, which stimulates Th17 lymphocytes, play an important role in the induction of excessive epidermal proliferation and inflammation in psoriasis. Cytokines in psoriasis are also secreted by cells of the epidermis and dermis, such as keratinocytes, DCs, fibroblasts and endothelial cells. Most of the cytokines of pathogenetic importance in psoriasis act through Janus-activated kinases (JAKs), which also play a role in the aetiopathogenesis of many other diseases, including inflammatory diseases and autoimmune diseases. They are part of the JAK–signal transducer and activator of transcription (STAT) signalling pathway. To date, four major JAK kinases have been described in the literature: JAK1, JAK2, JAK3 and TYK2 [2,6]. Thus, the entry of pro-inflammatory cytokines that play an important role in the aetiopathogenesis of psoriasis into the circulation can lead to pathological changes in distant organs, especially the cardiovascular system, and negatively affect glucose and lipid metabolism [2].

Psoriasis is an autoimmune inflammatory disease accompanied by an increased frequency of respiratory comorbidities that contribute to skin inflammation [7,8]. One of the most common symptoms accompanying psoriasis is pruritus, occurring in 64–98% of patients. Pruritus is usually confined to the affected skin, but 20–30% experience pruritus on unchanged skin, and some suffer from generalized pruritus. Pruritus has been shown to significantly impair the quality of life of patients with psoriasis [9].

Depending on the severity of the disease, local and systemic treatment is used. For patients with mild psoriasis, the mainstay of treatment remains topical agents, including tar, anthralin, corticosteroids, vitamin D3 analogues, calcineurin inhibitors and keratolytic agents such salicylic acid and urea. Another method used in the treatment of psoriasis is phototherapy, primarily narrow-band UVB, UVA or PUVA therapy, a combination of UVA light therapy and orally administered psoralen [3,6].

For many years, systemic treatments have included retinoids, cytostatic drugs, immunosuppressants, phosphodiesterase 4 inhibitors and, increasingly, biologics, which have been used with good therapeutic effect. The American Academy of Dermatology–National Psoriasis Foundation now even recommends biologic drugs as a first-line option due to their efficacy and safety profiles in the treatment of moderate-to-severe plaque psoriasis [4]. These biologics include inhibitors of tumour necrosis factor α (TNF-α), IL-12, IL-13, IL-17 and IL-23 [4]. Clinical trials of new antipsoriatic drugs are also under way focused on Retineic acid receptor-related orphan nuclear receptor gamma (RORγt) and JAK inhibitors, antagonists of the IL-36 receptor, rho-kinase (ROCK) inhibitors, sphingosine 1-phosphate (S1P) agonists and aryl hydrocarbon receptor (AhR) agonists [6,10].

Currently, a very promising field to help treat psoriasis is nanodermatology. Nanodermatology is a multidisciplinary discipline based on the interaction of many scientific fields. Solutions based on nanodermatology seem promising for the comprehensive and multidirectional treatment of psoriasis [11].

Recent studies have demonstrated the important role of sirtuins (SIRTs; silent information regulators) in numerous metabolic pathways in cells related to inflammation, tumorigenesis, as well as the cell cycle, DNA repair and proliferation, positioning them as an important element in the pathogenesis of many diseases, including psoriasis [9]. Hence, additional research on SIRTs as a possible point of entry for psoriasis pharmacotherapy is crucial.

## 2. SIRTs

SIRTs are an evolutionarily conserved group of enzymes involved in the post-translational modification of proteins including deacetylation, polyADP-ribosylation, demalonylation and lipoamidation. They exist in many cells and in a variety of organisms. Their names are derived from the Sir2 (silent information regulator 2) gene, first identified in cells of the yeast *Saccharomyces cerevisiae*. SIRTs are involved in the recombination of ribosomal DNA and participate in gene silencing and DNA repair [12]. They are involved in a number of important biological processes, such as transcriptional regulation, maintenance of chromosome stability, DNA repair, lipid and carbohydrate metabolism and the regulation of oxidative stress levels. SIRTs are deacetylases that remove acetyl groups from lysine residues of many proteins in a nicotinamide adenine dinucleotide (NAD^+^)-dependent reaction. In addition, they can function as mono-ADP ribosyl transferases, which perform a post-translational modification of proteins by mono-ADP ribosylation [12].

Human SIRT1 is the closest evolutionary homolog of the yeast Sir2 protein. In humans, there are seven known homologues of SIRT1, which differ in their localisation in the cell. SIRT1 and SIRT2 are present in both the cell nucleus and cytoplasm, while SIRT3, SIRT4 and SIRT5 are mitochondrial proteins. SIRT6 and SIRT7, on the other hand, are nuclear proteins [12].

**Figure 1:**
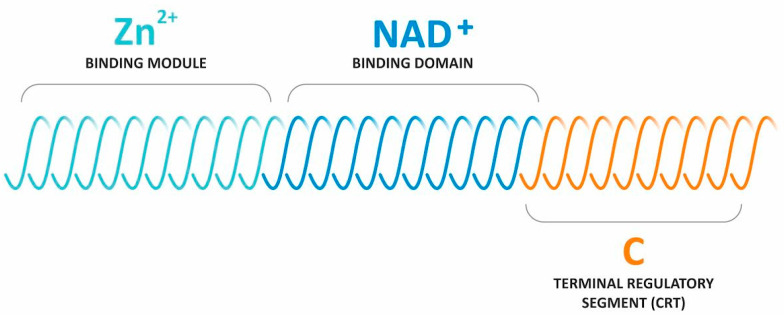
Schematic diagram of sirtuin’s structure.

SIRT1 has the ability to regulate inflammation-related signalling pathways and inhibit mitochondrial reactive oxygen species (ROS), oxidative stress, mitochondrial DNA mutation and mitochondrial damage, which in turn inhibits pancreatic β-cell damage, reducing the incidence of diabetes, obesity, insulin resistance and hepatic steatosis [13]. SIRT1, SIRT2 and SIRT6 affect metabolism and longevity by regulating the nuclear factor κB (NF-κB) and fatty acid β-oxidation signalling pathways [14]. SIRT3 inhibits proliferative capacity, promotes β-oxidation of fatty acids and activates key enzymes of the electron transport chain and urea cycle [15]. SIRT4 and SIRT5 activate the pyruvate dehydrogenase (PdH) complex, succinate dehydrogenase and the glutamate dehydrogenase (GdH) complex, which regulate cellular metabolism [16]. SIRT7 plays a role in the release of inflammatory cytokines, avoidance of DNA damage repair, adaptation to environmental challenges and cell survival [17]. A schematic diagram of sirtuin’s structure is shown in Figure 1.

## 3. The Role of SIRTs in Psoriasis

In recent years, SIRTs have been shown to be involved in a number of cellular pathways related to ageing, inflammation, oxidative stress, epigenetics, tumorigenesis, the cell cycle, DNA repair and proliferation, positioning them as an important part of the pathogenesis of many diseases, including psoriasis [12]. The production of inflammatory factors and immune cell differentiation processes are regulated by SIRT via deacetylation. A growing body of scientific evidence suggests that SIRTs participate in several metabolic pathways of cells and different inflammatory processes. Consequently, SIRTs may play an important role in the pathogenesis of inflammatory diseases, including psoriasis, affecting a variety of processes in the disease [18].

SIRT1 has been shown to promote human keratinocyte differentiation [19] and to inhibit keratinocyte proliferation [20]. SIRT1 also inhibits TNF-α transcription via deacetylation, which can lead to a reduction in the expression of inflammatory cytokines through this pathway [21]. SIRT1 overexpression reduces the expression of TNF-α, IL-1β and IL-8 [22]. In contrast, SIRT6, in the presence of NAD^+^, can increase TNF-α protein synthesis. Regulation of SIRT1 or SIRT6 activity can affect TNF-α levels [23].

Changes in SIRT expression in skin biopsy specimens from patients with psoriasis have been reported. In particular, significantly reduced SIRT1 expression in skin samples from patients with psoriasis compared with controls has been noted [24]. D’Amico et al. [24] reported that almost all nuclei in the epithelial layer of skin samples from healthy controls are positively stained for SIRT1, while in psoriatic samples the staining was rare and of negligible intensity. As reported by Fan et al. [25], in psoriasis there is a significant reduction in the expression of SIRT1, SIRT2, SIRT3, SIRT4 and SIRT5, and an increase in the expression of SIRT6 and SIRT7. The authors also revealed that SIRTs are mainly localised in the epithelial layer, which may mean that keratinocytes, as antigen-presenting cells, may primarily trigger an immune response rather than lymphocytes located subcutaneously.

**Figure 2:**
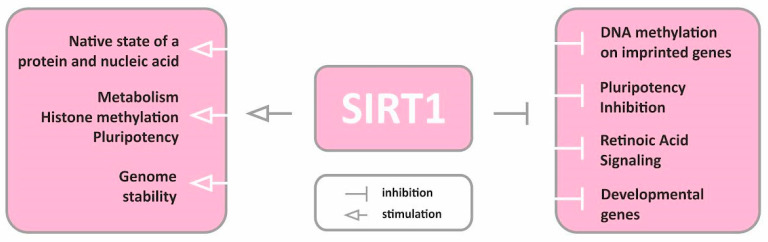
Multidirectional action of sirtuin 1.

A trial involving a SIRT1 activator (SRT2104) to treat patients with moderate-to-severe psoriasis has shown promising results with a satisfactory safety profile. SRT2104 significantly reduces the expression of IL-17- and TNF-α-responsive genes and genes involved in keratinocyte differentiation. Such reports warrant further investigation of new therapeutic options using SIRT activators for the treatment of psoriasis [26]. To date, most studies on the role of SIRTs in psoriasis have focused on SIRT1. The multidirectional action of sirtuin 1 is shown in Figure 2.

### 3.1. SIRT1 and Oxidative Stress in Psoriasis

**Figure 3:**
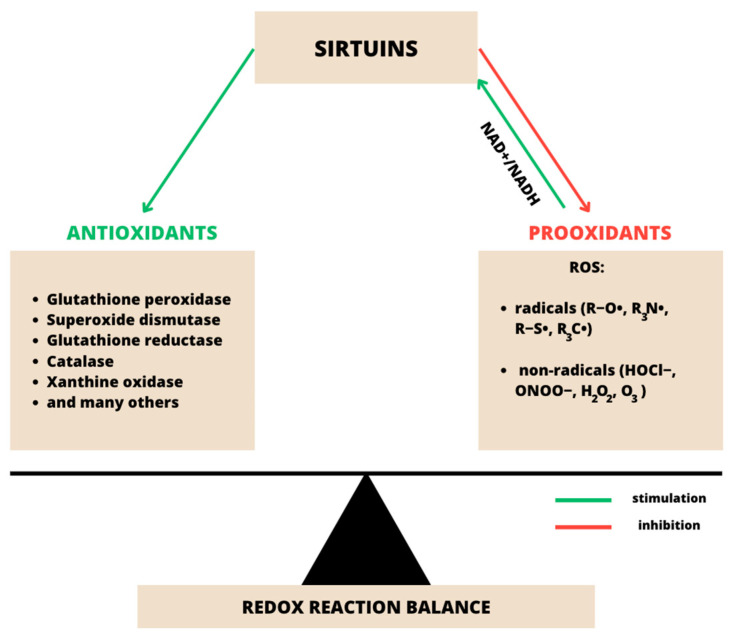
Diagram showing the maintenance of SIRT-mediated redox homeostasis. SIRTs help maintain redox homeostasis by balancing antioxidant enzymes and pro-oxidant radicals. SIRTs have been shown to regulate the expression and activity of antioxidant enzymes and the production of pro-oxidants. Pro-oxidants also affect the activity of SIRTs by altering the NAD^+^/NADH ratio, enabling a feedback loop that prevents cells from entering or remaining in a state of oxidative stress [26].

An imbalance between the amount of ROS and antioxidants leads to the generation of oxidative stress [27]. ROS primarily include the superoxide anion (O_2_·^−^), the hydroxyl radical (·OH), hydrogen peroxide (H_2_O_2_) and lipid radicals, which are by-products of cellular metabolism [28]. Some enzymes, such as superoxide dismutase (SOD), catalase (CAT) and glutathione peroxidases (GSH-px), act as antioxidants to protect cells from oxidative damage [29]. Figure 3 shows a schematic illustration of the role of SIRTs in maintaining redox balance [30].

ROS generation is a key step in the induction of oxidative stress in psoriasis, which exacerbates the course of psoriasis. ROS act as secondary messengers, increasing malondialdehyde (MDA), nitric acid (NO), ·OH and inducible nitric oxide synthase (iNOS), and decreasing SOD, CAT and GSH-px [31]. The increase in oxidation products activates Th1 and Th17 cells and keratinocytes through the mitogen-activated protein kinase (MAPK), NF-κB and JAK–STAT signalling pathways [32]. This leads to activation by Th11 and the emergence of a cascade of inflammatory cytokines and growth factors such as IFN-γ, IL-2, TNF-α, TNF-β, IL-6, IL-8 and vascular endothelial growth factor (VEGF), which are released due to keratinocyte activation. The aforementioned inflammatory mediators further activate T cells and mast cells, initiating a self-perpetuating loop that leads to excessive keratinocyte proliferation, neutrophil recruitment, vascular proliferation and persistent skin inflammation [33]. Angiogenesis, a marker of psoriatic pathogenesis, is also promoted by angiogenic mediators such as VEGF, TNF-α, IL-8 and IL-17 [34]. In addition, SIRT1 protects fibroblasts in patients with psoriasis from oxidative-stress-induced apoptosis and reduces MAPK signalling to restore both mitochondrial function and redox balance [35].

### 3.2. SIRT1 and the MAPK Pathway

MAPKs are a family of serine/threonine protein kinases involved in signal transduction, cell differentiation, proliferation, apoptosis and immune responses. Members of this family include extracellular signal-regulated kinase (ERK), c-Jun N-terminal kinase (JNK) and p38 MAPK [36]. AP-1 is a key eukaryotic transcription factor activated by three MAPK pathways and regulates various pro-inflammatory factors such as TNF-α, IL-6 and monocyte chemoattractant protein-1 (MCP-1). SIRT1 activation protects the heart from oxidative stress and the brain from alcohol-induced neurodegenerative processes via inhibition of the p38 MAPK pathway [37]. Studies have shown that activation of SIRT1 by carnosic acid protects mouse hepatocytes from oxidative-stress-induced cytotoxicity through the regulation of ERK1/2 [38]. There is increased activation of ERK1/2, p38 MAPK and JNK in psoriatic skin [39]. In fibroblasts from patients with psoriasis, SIRT1 activation has been shown to play an important role in restoring both mitochondrial function and redox balance through modulation of MAPK signalling [32].

### 3.3. SIRT1 and the STAT3 Pathway

In addition to the MAPK/AP-1 and NF-κB signalling pathways, STAT3 also plays an important role in psoriasis. STAT3 is involved in the regulation of basic biological processes such as cell proliferation, differentiation, oncogenesis, survival and apoptosis [40]. In resting cells, STAT3 is located in the cytoplasm. Upon stimulation by ROS, STAT3 is activated by tyrosine 705 (Tyr705) phosphorylation, enters the nucleus and regulates gene expression. STAT3 is mainly phosphorylated by JAK and epidermal growth factor receptor kinase. However, Src and ERK may also be involved in STAT3 phosphorylation. In addition, MAPKs, ERKs and protein kinases have been shown to increase STAT3 gene transcription [41]. Therefore, MAPK and STAT3 synergistically promote the development of psoriasis. Numerous studies have shown that STAT3 is overactivated in psoriatic lesions, promoting keratinocyte hyperproliferation and the production of IL-6, IL-8, IL-22, IL-23 and IL-17. These cytokines then trigger Th17 and STAT3 signalling pathways, leading to ongoing inflammation [42]. There is convincing evidence that SIRT1 antagonises IL-22-induced STAT3 activity by deacetylating STAT3 and inhibiting gene expression, thereby suppressing keratinocyte proliferation and migration [43]. In addition, SIRT1 reduced lesion severity in an Aldara-induced psoriasis model by decreasing STAT3 activation. A significant decrease in SIRT1 and, conversely, an increase in STAT3 were demonstrated in a mouse model of psoriasis compared with healthy mice [26].

### 3.4. SIRT 1 and the NF-κB Pathway

**Figure 4:**
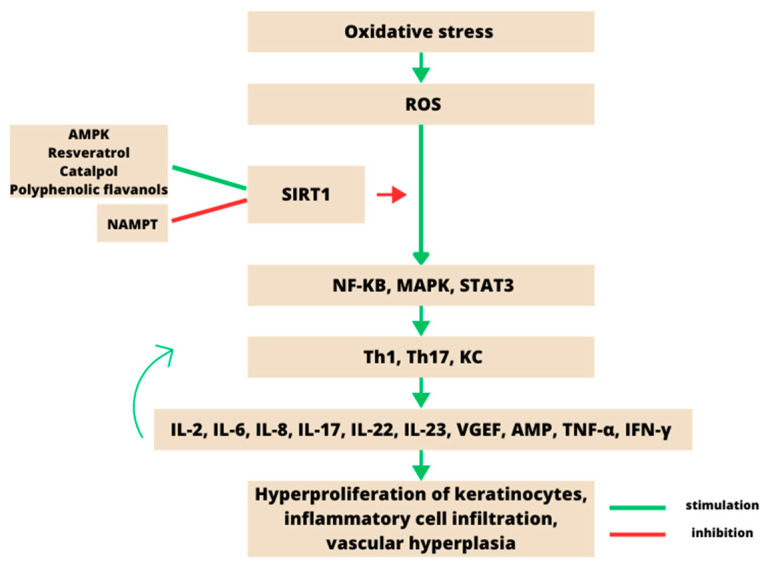
The effects of oxidative stress on psoriasis. An increase in oxidation products formed under oxidative stress leads to activation of Th1, Th17 cells and keratinocytes (KC). This occurs through the MAPK, NF-κB and JAK-STAT pathways, resulting in overproduction of IL-17, IL-22, IL-23, TNF-α, IFN-γ, IL-2, AMP, IL-6, IL-8 and VEGF. These inflammatory factors further activate Th1, Th17 and KC, forming a self-reinforcing loop, ultimately leading to excessive KC proliferation, vascular proliferation and inflammatory cell infiltration. Activation of SIRT1 inhibits the MAPK, NF-κB and STAT3 pathways, thereby reducing oxidative-stress-induced inflammation [46].

SIRT1 inhibits NF-κB signalling by deacetylating the p65 subunit of NF-κB, leading to a reduction in the inflammatory response [44]. SIRT1 can inhibit NF-κB signalling through adenosine monophosphate (AMP)-activated protein kinase (AMPK), peroxisome proliferator-activated receptor-γ coactivator (PGC-1α) and peroxisome proliferator-activated receptor α (PPARα). At the same time, NF-κB reduces SIRT1 expression and signalling by ROS, IFN-γ and poly (ADP-ribose) polymerase 1 (PARP-1) under oxidative stress [45]. The role of sirtuin 1 in psoriasis is shown in Figure 4.

### 3.5. SIRT1 and the AMPK Pathway

There is a reciprocal correlation between SIRT1 and AMPK [47]. SIRT1 is dependent on AMPK, which is a sensor of the AMP/adenosine triphosphate (ATP) ratio [48]. AMPK activation leads to an increase in NAD levels, which in turn activates SIRT1 [49]. In addition, the deacetylation activity of SIRT1 can affect AMPK activity [50]. Metabolic disorders that affect AMPK activation in keratinocytes may play a role in the pathogenesis of psoriasis. AMPK activity is reduced in psoriasis-affected skin. The researchers also noted that specific AMPK inhibitors stabilise messenger RNAs (mRNAs) involved in the development of psoriasis [51]. D’Amico et al. [24] showed a significant reduction in AMPK levels in psoriatic skin and noted that the expression of the phosphorylated/active form of this enzyme is completely abolished in psoriasis. On the other hand, AMPK-stimulating drugs, such as glucagon-like peptide-1 (GLP-1) analogues, show potential in the treatment of psoriasis because of their anti-inflammatory effects [52]. Another interesting finding is that inhibition of dipeptidyl peptidase 4, leading to an increase in GLP-1, contributes to promoting repair activity in the skin, as demonstrated in vitro and in vivo [53].

### 3.6. SIRT1 and the Nicotinamide Phosphoribosyltransferase (NAMPT) Pathway

SIRT1 activity depends on NAD^+^ availability [54]. SIRT1 can be regulated by NAMPT [55], which catalyses the reaction of nicotinamide (NAM) with 5-phosphoryl succinate to yield nicotinamide mononucleotide (NMN), an intermediate in NAD synthesis [56].

Increased expression of NAMPT has been observed in skin samples from patients with psoriasis compared with healthy subjects [19]. This has also been replicated in other studies [57,58]. In addition, microarray and real-time polymerase chain reaction (PCR) analyses have shown an overexpression of NAMPT in peripheral blood mononuclear cells of patients with psoriasis [59]. NAMPT overexpression is thought to contribute mainly to the pathogenesis of psoriasis by exacerbating an autoinflammatory loop, leading to an increased production of cytokines and chemokines by keratinocytes and promoting the recruitment of inflammatory cells from the blood [60].

### 3.7. SIRT2

The role of SIRT2 in psoriasis has not been extensively explored. SIRT2 has a role in regulating pyruvate kinase M2 (PKM2) activity in Th17-mediated inflammatory responses. In a mouse model of psoriasis, researchers observed decreased levels of SIRT2 and increased activation of PKM2 through the acetylation and phosphorylation of STAT3 [61]. They found that direct interaction between SIRT2 and non-acetylated PKM2 inhibits STAT3 phosphorylation. As a result, mice lacking SIRT2 exhibited more pronounced psoriasis. When SIRT2 was overexpressed or PKM2 was pharmacologically blocked, there was a reduction in the disease. A cytometric analysis of skin tissues from mice with blocked SIRT2 showed an increased number of Th17 cells. Ex vivo studies showed that SIRT2 deficiency accelerated Th17 cell differentiation while inducing IL-17A and IL-22 production. These findings suggest that the PKM2-mediated deacetylation of SIRT2 may be an effective therapeutic option for psoriasis [61]. Preliminary experimental studies appear to be promising, but an evaluation of the role of SIRT2 modulation in psoriasis therapy requires clinical trials.

### 3.8. SIRT3

The role of SIRT3 in psoriasis is very poorly understood. Oxidative stress leads to cellular dysfunction, and mitochondria are a major target for the toxicity of many chemicals [62]. Mitochondria can eliminate defective proteins and organelles through autophagy, a process termed mitophagy [63]. Impaired mitophagy leads to increased levels of cellular ROS and enhances pro-oxidant reactions [64]. In 2023, Yanli et al. [65] observed that SIRT3 is downregulated in mouse models of psoriasis. In contrast, SIRT3 overexpression activates parkin-dependent mitophagy through deacetylation of the traction factor FOXO3a to eliminate inflammation, oxidative stress and excessive cell proliferation in psoriasis. This discovery highlights the role of SIRT3 deficiency in psoriasis, and mitophagy mediated by SIRT3 may be a new therapeutic target for psoriasis treatment.

### 3.9. SIRT5

To date, there have been few studies on the role of SIRT5 in psoriasis. In epidermal keratinocytes, SIRT5 reduces cell proliferation and inflammation and improves the function of the damaged skin barrier by inhibiting the ERK/STAT3 pathway [66]. Ceramides (CERs) synthesised in epidermal keratinocytes are major components of the extracellular lipid matrix and are involved in maintaining normal barrier function [67]. Fatty acid elongases (ELOVL1 and ELOVL4) are involved in the production of very-long-chain CERs [68]. Wang et al. [66] showed that SIRT5 overexpression leads to decreased proliferation and expression of inflammatory factors, as well as increased amounts of ELOVL1 and ELOVL4, filaggrin, loricrin and aquaporin-3 in keratinocytes, indicating a protective role for SIRT5 in psoriasis. It has been confirmed that the above effect is achieved via SIRT5′s effect on ERK/STAT3. An increase in SIRT5 expression leads to a decrease in ERK and STAT3. STAT3 plays an important role in the development of psoriasis [69], excessive keratinocyte proliferation is directly related to the development of the disease, and STAT3 can promote cell proliferation and differentiation [70]. STAT3 inhibition is key to alleviating psoriasis symptoms, and studies have shown that ERK signalling affects STAT3 expression [71]. SIRT5 overexpression leads to reduced proliferation and reduced damage to skin barrier function, which is mediated by ERK/STAT3 in keratinocytes in response to IL-17A. SIRT5 promotes the acetylation of p65 to regulate the NF-κB pathway and has been implicated in reducing the expression of pro-inflammatory cytokines [72]. Researchers have identified a potential regulatory mechanism of SIRT5 that is involved in NF-κB modulation, involving the upregulation of ERK/STAT3 to decrease the amount of phosphorylated p65 [66].

## 4. SIRT-Activating Compounds

Resveratrol (RSV), or more specifically 3,5,4′-trihydroxystilbene, is the most potent activator of SIRT1 [73]. It is a naturally occurring polyphenol found mainly in grapes, berries and red wine [74]. RSV exhibits anti-inflammatory, antioxidant, antimicrobial and neuroprotective effects [75]. In addition, it has potent chemopreventive effects against skin, breast, prostate and lung cancers [76]. RSV passes through the cell membrane in three ways: passive diffusion, endocytosis through lipid rafts or via carrier-bound transport, binding to receptors such as integrin αvβ3 [77].

RSV participates in the regulation of innate and acquired immune responses by stimulating the activation of macrophages, T cells and natural killer (NK) cells, and cooperating in the inhibition of CD4+ and CD25+ T cells [78]. RSV can scavenge ROS, inhibit cyclooxygenase (COX) and activate multiple anti-inflammatory pathways, including SIRT1 [79]. Upon entering the cell, RSV activates SIRT1, leading to the inhibition of RelA acetylation, and stimulates inhibitor protein-B (IkB) degradation, which in turn reduces NF-kB-induced TNF-α, IL-1, IL-6, matrix metalloproteinase (MMP) and COX-2 production [77]. With its ability to activate SIRT1, RSV is able to alleviate inflammatory symptoms in experimental models of autoimmune diseases such as type I diabetes, encephalomyelitis and rheumatoid arthritis. Activation of SIRT1 by RSV leads to the inhibition of acetylation of p65/RelA, a member of NF-κB that plays a major role in regulating leukocyte activation and inflammatory cytokine signalling. This mechanism reduces the expression of NF-κB-induced inflammatory factors such as TNF-α, interleukin (IL)-1, IL-6, MMP-1 and MMP-3 and COX-2 [80].

In advanced psoriasis, activated DCs produce the key psoriasis-related cytokines IL-12 and IL-23 [81], which stimulate Th1 and Th17. As a result, there is an increase in the Th1-mediated expression of IL-12, IFN and IL-17 by Th17 cells [82]. Increased levels of these cytokines lead to disease progression by activating keratinocytes, promoting cell proliferation and differentiation [20]. In vitro studies have shown that RSV reduces the production of inflammatory cytokines and promotes keratinocyte apoptosis through activation of SIRT1. RSV controls keratinocyte death through signalling pathways mediated by SIRT1 and serine/threonine kinase (Akt). RSV is associated with increased expression and activation of SIRT1, leading to increased deacetylation of p53 and decreased phosphorylation of Akt. This protein plays an important role in regulating cell survival and proliferation. RSV-induced keratinocyte cell death is regulated by the SIRT1/phospho-Akt-dependent signalling pathway, confirming that it may have applications in psoriasis therapy [83].

Another study showed that RSV inhibits the proliferation of normal epidermal keratinocytes by blocking aquaporin 3 (AQP3), an important regulator of cell survival. This inhibition results from the activation of SIRT1, which leads to increased activation of the aryl hydrocarbon receptor nuclear translocator (ARNT), resulting in the dephosphorylation of growth factor-related signalling kinase (ERK) and preventing AQP3 activation [15]. A mouse model of imiquimod-induced psoriasis showed that RSV can ameliorate psoriasis-induced damage by reducing the thickness of the skin and downregulating the mRNA expression levels of IL17 and IL19, which are key cytokines in the development of the disease. It has also been observed that RSV increases the expression of the enzyme phosphoenolpyruvate carboxykinase 1 (PCK1) and TRIM63 protein, which are important for cell deletion and hypertrophy [84]. In addition, as shown in studies on skin keratinocytes, RSV can inhibit the UV-induced activation of NF-κB [85].

Sebum, produced by the sebaceous glands, consists mainly of triglycerides and fatty acids [86]. Sebaceous gland lipids play a role in pro- and anti-inflammatory signalling and affect immune cell function [87]. Functional damage to sebaceous glands plays a role in the pathogenesis of many inflammatory skin diseases, including psoriasis [88]. It is noteworthy that a reduced amount of SIRT1 is also found in the sebaceous glands of patients with psoriasis [89]. RSV is an activator of the AMPK–SIRT1 pathway, improving lipid accumulation and reducing inflammation in human keratinocytes, highlighting the importance of SIRT1′s anti-inflammatory properties in sebaceous glands [85].

Other polyphenolic plant flavanols, such as quercetin, apigenin, catechin, epicatechin, theobromine, curcumin, soy isoflavones, sulforaphane, olivetol, isothiocyanates, piceatannol, cinnamon and fisetin, provide health benefits through SIRT1 activation [90,91].

Catalpol is an iridoid glucoside isolated from the roots of *Rehmannia glutinosa*. Studies have shown that this substance has antidiabetic [92], anticancer [93] and antihyperglycaemic properties. Its antioxidant and anti-inflammatory effects have been confirmed in many experimental studies [94].

Zhou et al. examined the effects of catalpol on insulin resistance and adipose tissue inflammation in mice induced by a high-fat diet [94]. The oral administration of catalpol at a dose of 100 mg/kg for 4 weeks significantly reduced fasting glucose and insulin levels. Catalpol inhibited the influx of macrophages into adipose tissue, reducing the expression of pro-inflammatory cytokines in adipose tissue and increasing the expression of anti-inflammatory cytokines. At the same time, catalpol significantly inhibited c-Jun NH2-terminal kinase (JNK) and nuclear factor-kappa B (NF-κB) signalling pathways in adipose tissue [94]. Bi et al. evaluated the effect of catalpol on H_2_O_2_-induced oxidative stress in a mouse model. The results showed that catalpol reduced intracellular ROS formation in mouse astrocytes. Catalpol reduced oxidative stress by preventing a decrease in the activity of antioxidant enzymes such as glutathione peroxidase and glutathione reductase, thereby protecting cells from a decrease in glutathione, an important component of protection against oxidative stress [95].

It has been shown that catalpol can alleviate psoriasis by affecting SIRT1 [96]. Liu et al. evaluated the effect of catalpol on the development of psoriasis in mice with imiquimod-induced psoriasis [97]. The animals were given catalpol for 8 days. Catalpol reduced erythema, inflammation and scaling in the affected skin area in the mice. In addition, catalpol increased SIRT1 expression, while it blocked NF-kB and MAPK inflammatory pathways. The authors obtained similar results in cell cultures of TNF-α-stimulated keratinocytes treated with different concentrations of catalpol [96]. Xiong et al. investigated the signalling mechanisms underlying the catalpol-induced activation of SIRT1 and inhibition of endoplasmic reticulum stress in a rat model of colitis. They found that catalpol inhibited the endoplasmic reticulum stress marker proteins ATF6, CHOP and caspase12. In addition, catalpol reduced colitis symptoms, including inflammatory cell infiltration and pro-inflammatory cytokines [97]. Zhang et al. studied the effect of catalpol on adriamycin-induced kidney damage in mice. The administration of catalpol to mice significantly reduced adriamycin-induced kidney lesions, especially in the podocytes [98].

Most of the studies on the effects of catalpol have been conducted on animal models, and only a few clinical trials have evaluated this drug’s effectiveness. The efficacy of catalpol in the treatment of colorectal cancer was evaluated in a group of 115 patients with colorectal adenocarcinoma and 115 receiving a placebo. Patients in the catalpol-treated group had significantly reduced levels of carbohydrate antigen 19-9 (CA 19-9), carcinoembryonic antigen (CEA), matrix metalloproteinases-2 (MMP-2) and matrix metalloproteinases-9 (MMP-9) [99].

Topical therapy is one of the key methods used to treat skin diseases. This form of drug application allows higher drug concentrations to be achieved in the skin [100]. The topical application of resveratrol is a safe therapeutic strategy for treating plaque psoriasis. However, the low permeability of biomolecules through psoriatic skin and the inflammation occurring in lesional skin pose major challenges to developing an effective and safe form of topical application of the drug [100]. Polymeric micelles show many advantages over other new drug delivery systems, including an increased penetration of the drug into deeper layers of the skin, as well as a gradual and prolonged release of the drug from the polymeric micelles and a better solubility of the drug in the skin [100]. Khurana and his team [101] developed polymeric micelles that were filled with resveratrol and then transformed them into a gel. The study confirmed the significant advantage of the polymeric micelle gel over traditional gel. A reduction in PASI score, serum cytokine levels and hyperkeratosis was observed in patients treated with the polymer micelle gel containing resveratrol. Another study used a nanocarrier gel with resveratrol vesicles. A group of subjects administered the gel with a concentration of 73.76 ± 2.46% resveratrol experienced significant improvements in skin condition. Reduced erythema, desquamation and improved overall skin condition were observed, with minimal changes in mRNA gene expression for inflammatory cytokines [102].

## 5. Conclusions

SIRTs are a family of deacetylase-like proteins that exhibit anti-inflammatory and immunomodulatory effects, which may be important in the treatment of psoriasis. Activation of SIRT1 counteracts oxidative-stress-induced damage and restores redox balance by inhibiting the MAPK, NF-κB and STAT3 pathways, thereby alleviating the course of psoriasis. In this article, we have reviewed the mode of action of SIRTs in psoriasis and the substances that may mediate their activation or suppression. This information may be applicable when evaluating potential treatments for psoriasis. This review should draw the attention of physicians and scientists to the importance of SIRTs in dermatology and provide a basis and impetus for future discussions, research and pharmacological discoveries to modulate the action of these enzymes. Based on their activity, SIRT1–SIRT5 agonists and SIRT6 and SIRT7 inhibitors may represent new therapeutic options for the treatment of psoriasis, but more studies are needed to confirm this eventuality. SIRT-activating substances, especially RSV with pleiotropic effects in psoriasis, also deserve attention. In conclusion, SIRTs show potential therapeutic effects in psoriasis by reducing the inflammatory response and regulating skin cell proliferation and immunomodulation. Despite the promising data, additional studies are needed to fully understand the mechanism of action of SIRTs and to evaluate their therapeutic efficacy in patients with psoriasis.

## Data Availability

Not aplicable.
